# Stress-Related Mental Health Symptoms in Coast Guard: Incidence, Vulnerability, and Neurocognitive Performance

**DOI:** 10.3389/fpsyg.2017.01513

**Published:** 2017-09-14

**Authors:** Richard J. Servatius, Justin D. Handy, Michael J. Doria, Catherine E. Myers, Christine E. Marx, Robert Lipsky, Nora Ko, Pelin Avcu, W. Geoffrey Wright, Jack W. Tsao

**Affiliations:** ^1^Department of Veterans Affairs, Syracuse Veterans Affairs Medical Center Syracuse, NY, United States; ^2^Department of Psychiatry, State University of New York Upstate Medical University Syracuse, NY, United States; ^3^Rutgers Biomedical Health Sciences, Stress and Motivated Behavior Institute, Rutgers University Newark, NJ, United States; ^4^United States Coast Guard Washington, DC, United States; ^5^Department of Veterans Affairs, VA New Jersey Health Care System East Orange, NJ, United States; ^6^Department of Pharmacology, Physiology & Neuroscience, Rutgers Biomedical Health Sciences, Rutgers University Newark, NJ, United States; ^7^Veterans Affairs Mid-Atlantic Mental Illness, Research Education and Clinical Center, Durham Veterans Affairs Medical Center Durham, NC, United States; ^8^Department of Psychiatry and Behavioral Sciences, Duke University School of Medicine Durham, NC, United States; ^9^Department of Neurosciences, INOVA Health System Fairfax, VA, United States; ^10^Program of Neuroscience, Graduate School of Biomedical Sciences, Rutgers University Newark, NJ, United States; ^11^Neuromotor Sciences Program, Temple University Philadelphia, PA, United States; ^12^Department of Neurology, University of Tennessee Health Science Center Memphis, TN, United States; ^13^Department of Neurology, Memphis Veteran Affairs Administration Medical Center Memphis, TN, United States; ^14^Children's Foundation Research Institute, Le Bonheur Children's Hospital Memphis, TN, United States

**Keywords:** PTSD, depression, military personnel, temperament, personality

## Abstract

U.S. Coast Guard (CG) personnel face occupational stressors (e.g., search and rescue) which compound daily life stressors encountered by civilians. However, the degree CG personnel express stress-related mental health symptoms of posttraumatic stress disorder (PTSD) and major depressive disorder (MDD) is understudied as a military branch, and little is known concerning the interplay of vulnerabilities and neurocognitive outcomes in CG personnel. The current study addressed this knowledge gap, recruiting 241 active duty CG personnel (22% female) to assess mental health, personality, and neurocognitive function. Participants completed a battery of scales: PTSD Checklist with military and non-military prompts to screen for PTSD, Psychological Health Questionnaire 8 for MDD, and scales for behaviorally inhibited (BI) temperament, and distressed (Type D) personality. Neurocognitive performance was assessed with the Defense Automated Neurobehavioral Assessment (DANA) battery. Cluster scoring yielded an overall rate of PTSD of 15% (95% CI: 11–20%) and 8% (95% CI: 3–9%) for MDD. Non-military trauma was endorsed twice that of military trauma in those meeting criteria for PTSD. Individual vulnerabilities were predictive of stress-related mental health symptoms in active duty military personnel; specifically, BI temperament predicted PTSD whereas gender and Type D personality predicted MDD. Stress-related mental health symptoms were also associated with poorer reaction time and response inhibition. These results suggest rates of PTSD and MDD are comparable among CG personnel serving Boat Stations to those of larger military services after combat deployment. Further, vulnerabilities distinguished between PTSD and MDD, which have a high degree of co-occurrence in military samples. To what degree stress-related mental healthy symptoms and attendant neurocognitive deficits affect operational effectiveness remains unknown and warrant future study.

## Introduction

Serving as one of the five armed forces of the United States, the Coast Guard (CG) is unique in having domestic and international missions. The CG conducts 11 missions: aids in navigation, defense readiness, drug interdiction, ice operations, living marine resources, marine environmental protection, marine safety, migrant interdiction, port security, search and rescue, and other law enforcement. In Boat Stations, CG military service bears resemblance to civilian law enforcement: high risk missions, a high degree of unpredictability regarding when a mission is required and its degree of difficulty. Like firefighters, many CG personnel serving at Boat Stations have rotating shift schedules mixing on-duty and off-duty days on a weekly basis. Thus, active duty at Boat Stations represents a mixture of military and civilian life experiences.

Military stressors have long been associated with mental health problems, particularly posttraumatic stress disorder (PTSD) and major depressive disorder (MDD). Accordingly, the increased risk for PTSD and MDD among the larger services arising from deployment and attendant combat service are well-documented (Shen et al., 2010; Mayo et al., 2013; Hines et al., 2014; Stander et al., 2014; Mustillo et al., 2015). In parallel, studies of PTSD and MDD in civilian police and firefighters focus on critical incidents such as World Trade Center (Perrin et al., 2007; Biggs et al., 2010; Bowler et al., 2010) and Hurricane Katrina (Tak et al., 2007; West et al., 2008). These studies are in keeping with the view extreme stressors and traumas have a direct effect on PTSD expression.

CG personnel are an understudied population; prevalence rates for PTSD and MDD are largely unknown. A comparison between the different services may be made using hospitalizations for stress-related mental health disorders as a guide. Surveillance of hospitalizations for PTSD finds rates are highest for Army and Marines, lower among the Navy and Air Force, and lower still for CG (Armed Forces Health Surveillance Center, 2013). Similarly, rates of hospitalizations for MDD among CG personnel are about half that of Army, and similar to Air Force, Navy, and Marines (Armed Forces Health Surveillance Center, 2013). Active duty military are notoriously averse to seeking mental health support (Iversen et al., 2011), thus hospitalizations may severely underestimate actual prevalence.

In diathesis models of PTSD and MDD, trauma serves a necessary but insufficient role in etiology. Inherent vulnerabilities interact with risk factors in the development of stress-related mental health problems. Personality and dispositional factors serve as one source of vulnerability. For instance, behaviorally inhibited (BI) temperament, characterized by extreme withdrawal in the face of social and non-social challenges, potentiates aversive learning in civilian and veteran samples and is associated with expression of PTSD in veterans (Myers et al., 2012a,b). Further, distressed (Type D) personality, which is typified by high levels of negative affect (NA) and social inhibition (SI), is a vulnerability factor of poor physical and mental health outcomes, including PTSD and MDD.

Beyond the development and expression of mental health difficulties, there is growing concern that stress may degrade neurocognitive performance and adversely affect operational performance. Neurocognitive deficits have been reported after repetitive military environmental exposures (e.g., blasts; Luethcke et al., 2011), stress-related mental disorders (Marx et al., 2009; Luethcke et al., 2011; Haran et al., 2013), or mild traumatic brain injury (mTBI; Spira et al., 2014; Dretsch et al., 2015). The Defense Automated Neurobehavioral Assessment (DANA) battery, developed as a clinical decision support tool, consists of a battery of neurocognitive tests as well as psychological assessments for field screening of PTSD and MDD (Lathan et al., 2013; Spira et al., 2014). Using the DANA, multiple experiences of lifetime TBI were found to affect simple reaction time upon second testing, procedural reaction time and code substitution, but only in models unadjusted for PTSD and MDD (Spira et al., 2014). Although suggestive, the impact of PTSD and MDD on neurocognitive functioning was not separately evaluated.

The CG population in Boat Stations has a blend of military service and extensive non-military (but on-call) time, which offers the opportunity to examine the impact of different sources of trauma on PTSD. Therefore, the objectives of the current study were to: Objective (A) describe the demographic characteristics of the CG sample and responses to mental health and personality scales, Objective (B) determine prevalence of PTSD and MDD in CG personnel serving Boat Stations, Objective (C) examine vulnerability and risk factors for PTSD and MDD, and Objective (D) compare the neurocognitive performance of groups as a function of PTSD and MDD.

We expected rates of PTSD to be equivalent with respect to military or non-military trauma. Further, we expected personality scales to provide unique or distinguishable contributions to stress-related mental health difficulties, with BI temperament predicting PTSD and Type D personality, with its emphasis on negative affectivity, predicting general distress. We further expected MDD and its psychomotor slowing to be reflected in deficits in reaction time tasks and attentional processing (Thomas et al., 1999; Naismith et al., 2003), whereas PTSD would be associated with memory difficulties (Honzel et al., 2014).

## Materials and methods

A cross-sectional study was conducted in active duty CG personnel serving at Boat Stations. To gain insight into the source of traumatic stress symptoms, personnel were asked to complete the two version of the PTSD Checklist (PCL), with prompts related to military (PCL-M) and non-military (PCL-NM) traumatic experiences. Symptoms of MDD were assessed with the Personal Health Questionnaire (PHQ-8). In addition, personnel were administered scales assessing BI and Type D personality. Neurocognitive performance was assessed with the DANA.

### Participants and recruitment

Data were obtained as part of a larger study *Cognitive Assessment in Coast Guard Personnel: Neuroendocrine, Genetic and Epigenetic Correlates*, with data collection occurring during the period of 2013–2015. Recruitment was conducted by designated experimenters at eight CG Boat Stations (Golden Gate, St. Petersburg, New York, San Francisco, Seattle, Port Canaveral, New Orleans, and Port Lauderdale). Active duty military personnel (*N* = 241; 52 females and 189 males) were recruited at the beginning of the Stations' off-going and on-coming duty section period, followed by individual consenting for those interested in participating. An additional 9 CG personnel were consented but were excluded due to substantially incomplete data sets. Designated ombudsmen ensured that potential participants understood that participation was voluntary and refusal to participate involved no penalty or loss of benefits within CG. CG personnel were not compensated for participation. Eligibility was contingent on not having a Deployment Limiting Medical Condition as defined in the Coast Guard Medical Manual, COMDTINST M6000.1F. This study was carried out in accordance with the recommendations of the United States Coast Guard with written informed consent from all subjects. All subjects gave written informed consent in accordance with the Declaration of Helsinki. The protocol was approved by the United States Coast Guard and Rutgers University Biomedical Health Sciences Institutional Review Boards.

### Measures

#### Posttraumatic stress symptoms

To capture sources of trauma symptoms, two versions of the PCL were administered: one with a military stress prompt (PCL-M) and one with a non-military prompt (PCL-NM), respectively indicating whether personnel should describe symptoms related to a stressful military or non-military experience. Except for the prompts, the questions were identical. However, the PCL-M was electronically administered on the DANA, whereas the PCL-NM was completed on paper. The PCL assesses symptoms severity in the past month from 17 items; each item is scored on a 5-point Likert scale (0–4) yielding a range of 17–85. The PCL has convergent reliability and validity with sensitivity and selectivity for the diagnosis of PTSD (using *DSM- IV* criteria). For PTSD, caseness was determined by cluster scoring, which requires a score of 3 or greater on: one Cluster B symptom, three Cluster C symptoms and two Cluster D symptoms (Keen et al., 2008; McDonald and Calhoun, 2010; Lee et al., 2014; Searle et al., 2015). Subclinical PTSD was determined based on meeting criteria for two out of the three symptom clusters.

#### Depressive symptoms

The Patient Health Questionnaire 8 (PHQ-8) was used to assess how often depressive symptoms were bothersome over the last two week period. Occurrence was rated “not at all,” “several days,” more than half the days', and “nearly every day.” Screening for caseness used two methods: symptom scoring which required endorsement of either depressed mood or anhedonia by “more than half the days” and at least 5 of the 8 symptoms to be present “more than half the days” (Kroenke et al., 2009), and aggregate scoring which classified MDD as No (0–4), Mild (5–9), Moderate (10–14), Moderately Severe (15–20), and Severe (>20), with a score of 10 or more as indicative of clinically significant MDD.

#### Concussion history

The DVBIC TBI Screening Tool (Terrio et al., 2011; Schwab et al., 2015) was used to assess present/lifetime mTBI status. Verbally and individually administered with each participant, the screening tool determines whether the participant experienced a head injury, whether the participant lost consciousness and for how long, and the degree to which current symptoms are attributable to head injury.

#### Type D personality

Distressed, or Type D personality, was assessed with the DS14 (Denollet, 2000), which includes two 7-question subscales capturing negative affect (NA) and social inhibition (SI). Responses on the DS14 use a 5-point Likert scale (0–4), ranging from “False” to “True.” To be classified as Type D personnel had to score >9.5 on each of the NA and SI subscales (Emons et al., 2007).

#### BI temperament

The Adult Measure of Behavioral Inhibition (AMBI) consists of 16 items probing aspects of BI temperament (Gladstone and Parker, 2005). Items assess the degree to which behaviors are exhibited in social and non-social situations on a 3-point Likert scale (ranging from “No/hardly ever” to “Yes/most of the time”). Total scores range from 0 to 32. Those with scores above 15.5 were classified as BI.

#### Combat exposure

Previous combat exposure was assessed with the Combat Exposure Scale (CES; Keane et al., 1989). The CES is a self-report survey of combat experiences. Respondents were classified as either having previous combat exposure or not.

#### Sleep quality

The Pittsburg Sleep Quality Inventory (PSQI; Buysse et al., 1989) is composed of 19 self-rated questions and 5 questions rated by a bed partner or roommate (only the self-rated items are used in scoring the scale). The self-administered scale contains 15 multiple-choice items that inquire about frequency of sleep disturbances and subjective sleep quality; and 4 write-in items that inquire about typical bedtime, wake up time, sleep latency and sleep duration.

#### Neurocognitive tests

The DANA is a portable ruggedized computer device loaded with a battery of neurocognitive tests and psychological scales (Lathan et al., 2013). The “standard” version includes assessments of: Simple Reaction Time, Code Substitution: Learning, Procedural Reaction Time, Spatial Discrimination, Go/No-Go, Code Substitution: Recall, Matching to Sample, Sternberg Memory Search, and a second Simple Reaction Time measure. These tasks are followed by psychological scales: PCL-M and PHQ-8.

The DANA has three versions: “standard,” “rapid,” and “brief,” which are distinguished by the number of the tests and batteries administered. For the first 31 participants the “rapid” was administered, for all others “standard.” For these 31 participants, PHQ-8 data is missing; PCL-M was administered in paper form. Also for these 31 participants, neurocognitive testing was restricted to Simple Reaction Time, Procedural Reaction Time, and Go/No-Go.

### Analytic approach

1. Objective A. Demographic and service-related characteristics of the study sample were assessed using chi-square tests.
2. Objective B. Prevalence of PTSD was compared and contrasted for the PCL-M and PCL-NM using symptom and aggregate scoring methods, with agreement in incidence rates for PTSD caseness captured by each measure assessed using Cohen's κ. Prevalence rates for MDD were also assessed as a function of symptom and aggregate scoring methods.
3. Objective C. To explore risk and vulnerability factors for stress-related mental health symptoms, multinomial regression models were constructed to identify features of the study sample that predicted overall rates of subclinical/clinical PTSD and mild depression/MDD. In model building, we included stable preexisting personal characteristics (age, gender, BI, and Type D), preexisting experiential characteristics (number of concussions), and experiential characteristics of military service (deployment and combat exposure). Total scores for the PCL (highest score on either the PCL-M or PCL-NM) and PHQ-8 were also entered as predictors in models for MDD and PTSD, respectively. Unless otherwise noted, asymptomatic symptom groups were always used as the reference category for symptom groups. Odds ratios (OR) and 95% confidence intervals (CI) are reported for significant predictors.
4. Objective D. Finally, neurocognitive performance on the DANA was assessed using multivariate analysis of covariance (MANCOVA). Personnel were stratified according to stress-related mental health symptoms, resulting in a subclinical/clinical PTSD symptom group (PTSD+), mild depression/MDD symptom group (MDD+), co-morbid PTSD/MDD symptom group (Co-Occurring+), and no symptoms (Asymptomatic). Throughput scores for each of the nine DANA battery assessments served as dependent measures, with age, gender, and number of concussions entered as covariates. Descriptive discriminant analysis was used to follow-up a significant multivariate between-subjects effect, assessing linear combinations of neurocognitive assessments that maximized performance differences in symptom groups. Standardized canonical discriminant function coefficients generated by descriptive discriminant analysis were then used to create multivariate composite scores, as recommended by Harris (2001). The simplified composite scores for neurocognitive performance were entered as dependent measures in univariate ANCOVA, using symptom groups as the between-subjects variable and controlling for age, gender, and number of concussions. *Post-hoc* analyses for a significant main effect of symptom groups was analyzed using independent samples *t*-tests, corrected for inflated risk of Type 1 error using a Bonferroni correction.

All data analyses were conducted using SPSS version 23.0 for Windows. The rejection criterion for statistically significant results was set at *p* < 0.05 for all analyses, unless otherwise noted.

## Results

### Demographic features and psychometric properties

Recruitment within CG Boat Stations yielded a diverse group of participants. Demographic characteristics of the study sample are presented in Table 1. The overall sample contained a large number of female personnel (22%), and thus allowed for a comparison of gender differences on demographic features. Whereas the sample was generally young, with 65% younger than 30 years old, females were disproportionately younger with 91% younger than 30. A range of ranks volunteered, from enlisted personnel to officers; females in the sample were disproportionately lower in rank. With regard to sleep quality, a higher proportion of females reported greater sleeper disturbances. Lifetime history of concussion differed with a higher proportion of males previously experiencing a concussion than females.

**Table 1:** Demographic characteristics of the CG sample.

	**Overall**	**Male**	**Female**	**Probability (male vs. female)**
**Age**	***N*** = **241**	***N*** = **189**	***N*** = **52**	
< 25 years	74 (31%)	43 (23%)	31 (60%)	$\chi_{\left(2\right)}^{2}$ = 30.63, *p* < 0.001
25–29 years	82 (34%)	66 (35%)	16 (31%)	
>29 years	85 (35%)	80 (42%)	5 (9%)	
**Ethnicity**	***N*** = **241**	***N*** = **189**	***N*** = **52**	
White/Non-Hispanic	176 (73%)	140 (74%)	36 (69%)	$\chi_{\left(3\right)}^{2}$ = 1.55, *p* = 0.671
Black/Non-Hispanic	8 (3%)	7 (4%)	1 (2%)	
Hispanic	35 (15%)	25 (13%)	10 (19%)	
Other	22 (9%)	17 (9%)	5 (10%)	
**Education**	***N*** = **241**	***N*** = **189**	***N*** = **52**	
Some college or less	197 (82%)	157 (83%)	40 (77%)	$\chi_{\left(1\right)}^{2}$ = 1.03, *p* = 0.310
Bachelor's or higher	44 (18%)	32 (17%)	12 (23%)	
**Lifetime History of Concussion**	***N*** = **233**	***N*** = **183**	***N*** = **50**	
One	70 (30%)	64 (34%)	6 (12%)	$\chi_{\left(2\right)}^{2}$ = 17.50, *p* < 0.001
More than one	42 (18%)	37 (20%)	5 (10%)	
**Rank**	***N*** = **236**	***N*** = **185**	***N*** = **51**	
Cadet	7 (3%)	3 (2%)	4 (8%)	$\chi_{\left(4\right)}^{2}$ = 34.29, *p* < 0.001
Junior Enlisted (E1–E4)	119 (50%)	78 (42%)	41 (80%)	
NCO (E5–E6)	91 (39%)	86 (46%)	5 (10%)	
Senior NCO (E7–E9)	12 (5%)	12 (6%)	0	
Officer	7 (3%)	6 (3%)	1 (2%)	
**Deployment**	***N*** = **233**	***N*** = **184**	***N*** = **49**	
Previously Deployed	104 (45%)	94 (51%)	10 (20%)	$\chi_{\left(1\right)}^{2}$ = 0.61, *p* = 0.437
**Sleep Quality**	***N*** = **209**	***N*** = **162**	***N*** = **47**	
Poor Sleep Quality	118 (56%)	85 (52%)	33 (70%)	$\chi_{\left(1\right)}^{2}$ = 4.67, *p* = 0.031
**Combat Exposure**	***N*** = **232**	***N*** = **181**	***N*** = **51**	
Yes	20 (9%)	19 (11%)	1 (2%)	$\chi_{\left(1\right)}^{2}$ = 3.68, *p* = 0.055
**Type D**	***N*** = **239**	***N*** = **187**	***N*** = **52**	
Type D	58 (24%)	43 (23%)	15 (29%)	$\chi_{\left(1\right)}^{2}$ = 0.76, *p* = 0.384
**BI**	***N*** = **241**	***N*** = **189**	***N*** = **52**	
Inhibited	80 (33%)	62 (33%)	19 (37%)	$\chi_{\left(1\right)}^{2}$ = 0.06, *p* = 0.806

The reliability and intercorrelations of the PCLs, PHQ-8, DS-14, and AMBI are presented in Table 2. Strong positive correlations were apparent between the symptom checklists and the personality scales. Cronbach's α was in the acceptable range for all measures.

**Table 2:** Reliability and interrelatedness of scales.

	**1**	**2**	**3**	**4**	**5**
1 PCL-M	–				
2 PCL-NM	0.69^**^	–			
3 PHQ-8	0.70^**^	0.64^**^	–		
4 DS14	0.49^**^	0.56^**^	0.61^**^	–	
5 AMBI	0.37^**^	0.43^**^	0.33^**^	0.51^**^	–
Mean	25.71	28.95	4.80	19.53	14.17
St.Dev	9.83	11.65	4.67	8.62	5.69
Range	17–69	17–66	0–20	3–46	3–30
Chronbach's α	0.92	0.93	0.87	0.89	0.83

PCL-M, PTSD Checklist Military; PCL-NM, PTSD Checklist Non-Military; PHQ, Personal Health Questionnaire; DS14, Type D Scale; AMBI, Adult Measure of Behavioral Inhibition;

** *p < 0.01*.

### Prevalence of stress-related mental health symptoms

Administering two PCLs allowed for a comparison of caseness and symptoms emanating from military and non-military sources of trauma. Caseness for PTSD given the military prompt was 6% (15/241, 95% CI: 4–10%) and PTSD given the non-military prompt was 13% (32/241, 95% CI: 10–18%). The overall caseness for PTSD, defined as meeting criteria on either the PCL-M or PCL-NM, was 15% (37/241, 95% CI: 11–20%), which included 10 personnel that met criteria on both screening tools (see Table 3). McNemar's test revealed significantly more cases were identified with the PCL-NM than the PCL-M, χ^2^ = 9.48, *p* = 0.002. Significant agreement was evident between the PCL-M and PCL-NM as measured by Cohen's κ (κ = 0.37, *p* < 0.001). With regard to Subclinical PTSD, of those personnel not meeting diagnostic criteria for PTSD, 18% (36/204; 95% CI: 13–24%) were considered subclinical, with 6 cases identified by both screening tools.

**Table 3:** Rates of subclinical and PTSD Based on PCL-M and PCL-NM.

		**PCL-NM**			
		**No**	**Subclinical PTSD**	**PTSD**	**Total**
**PCL-M**	**No**	168	23	15	206/241 (85%)
	**Subclinical PTSD**	7	6	7	20/241 (8%)
	**PTSD**	3	2	10	15/241 (7%)
	**Total**	178/241 (74%)	31/241 (13%)	32/241 (13%)	241

*PCL-M, PTSD Checklist Military; PCL-NM, PTSD Checklist Non-Military*.

To facilitate comparisons with the literature, MDD was characterized using both aggregate and symptom scoring methods. Caseness of MDD using symptom scoring was 5% (12/232, 95% CI: 3–9%), whereas the more liberal aggregate scoring yielded 13% (31/232, 95% CI: 10–18%).

Prevalence of co-occurring PTSD/MDD was 19% (7/36, 95% CI: 10–35%) and 39% (14/36, 95% CI: 25–55%) based on symptom and aggregate scoring methods, respectively; caseness of MDD alone was 2.5% (5/196, 95% CI: 1–6%) and 9% (17/196, 95% CI: 5–14%) based on symptom and aggregate scoring methods, respectively. These rates are consistent with the high correlations between PHQ-8 total scores and PCL-NM and PCL-M total scores (see Table 2). When examining the co-occurrence of PTSD/MDD as a function of PCL, we observed rates of 20% (1/5, 95% CI: 4–63%) in those meeting criteria for PTSD on the PCL-M only, 29% (6/21, 95% CI: 14–50%) in those meeting criteria on the PCL-NM only, and 70% (7/10, 95% CI: 40–89%) in those meeting criteria on both the PCL-M and PCL-NM. However, given the small sample sizes in each of these groups, prevalence estimates may lack precision, as evidenced by the large 95% confidence intervals.

### Predictors of stress-related mental health symptoms

The results of the multinomial logistic regression are shown in Table 4. For Subclinical PTSD, PHQ-8 total score emerged as a significant positive predictor (OR: 1.25, 95% CI: 1.11–1.41), as did BI (OR: 3.41, 95% CI: 1.28–9.09). A similar pattern was apparent for factors predicting PTSD, with PHQ-8 total score (OR: 1.37, 95% CI: 1.21–1.54) and BI (OR: 4.54, 95% CI: 1.62–12.76) serving as significant positive predictors.

**Table 4:** Predictors of subclinical and clinical PTSD based on multinomial logistic regression.

	**PCL**						**PHQ-8**					
	**Subclinical PTSD**			**PTSD**			**Mild Depression**			**MDD**		
**Variable**	***B (SE)***	**OR**	**95% CI**	***B (SE)***	**OR**	**95% CI**	***B (SE)***	**OR**	**95% CI**	***B (SE)***	**OR**	**95% CI**
**Age**	−0.06 (0.04)	0.94	0.86–1.02	−1.93 (1.28)	0.95	0.87–1.03	0.06 (0.03)	1.06	0.99–1.14	−0.02 (0.06)	0.98	0.88–1.10
**Sex**												
Female	0.14 (0.53)	1.15	0.41–3.25	−0.88 (0.60)	0.42	0.13–1.36	1.11 (0.47)	3.04^*^	1.21–7.62	1.81 (0.65)	6.12^**^	1.70–21.95
Male (ref)												
**Lifetime history of concussion**												
One	−0.80 (0.55)	0.92	0.32–2.69	−0.65 (0.61)	0.52	0.16–1.74	−1.05 (0.50)	0.35^*^	0.13–0.93	0.43 (0.68)	1.54	0.41–5.84
More Than One	0.42 (0.58)	1.51	0.49–4.70	−0.18 (0.61)	0.83	0.25–2.72	−0.58 (0.53)	0.56	0.20–1.58	0.69 (0.71)	1.99	0.49–8.06
None (ref)												
**Combat Exposure**												
Yes	−0.94 (1.13)	0.39	0.04–3.58	0.57 (0.73)	1.76	0.42–7.34	0.18 (0.78)	1.19	0.26–5.44	1.85 (0.87)	6.38^*^	1.17–34.91
No (ref)												
**Type D**												
Type D	−0.51 (0.58)	0.60	0.19–1.88	−0.19 (0.57)	0.82	0.27–2.52	2.26 (0.52)	9.56^**^	3.46–26.40	2.38 (0.69)	10.81^**^	2.78–41.98
Non-Type D (ref)												
**BI**												
Inhibited	1.23 (0.50)	3.41^*^	1.28–9.09	1.51 (0.53)	4.54^**^	1.62–12.76	−0.21 (0.47)	0.81	0.32–2.04	−0.57 (0.69)	0.56	0.15–2.16
Non-Inhibited (ref)												
**PHQ-8 Total Score**	0.22 (0.06)	1.25^**^	1.11–1.41	0.31 (0.06)	1.37^**^	1.21–1.54						
**PCL Total Score**							0.09 (0.02)	1.09^**^	1.05–1.14	0.16 (0.03)	1.17^**^	1.11–1.23
**$R_{\text{CS}}^{2}$**	0.32						0.45					
**Model** χ^2^	$\chi_{\left(16\right)}^{2}$ = 84.44, *p* < 0.001						$\chi_{\left(16\right)}^{2}$ = 133.61, *p* < 0.001					

B, Logistic coefficient; SE, standard error; OR, odds ratio; CI, confidence interval; R2CS, Cox and Snell R2; ref, reference category; PHQ, Personal Health Questionnaire; PCL, PTSD Checklist; BI, Behavioral Inhibition. Significance levels:

* *p < 0.05*,

** *p < 0.01*.

Using aggregate scoring, the sample was divided into “Asymptomatic,” “Mild Depression,” and “MDD” groups. As shown in Table 4, multinomial logistic regression revealed higher PCL total scores (OR: 1.09, 95% CI: 1.05–1.14), male gender (OR: 3.04, 95% CI: 1.21–7.62) and Type D personality (OR: 9.56, 95% CI: 3.46–26.40) to be positive predictors of Mild Depression, whereas prior history of one concussion (compared to no concussion) was a negative predictor (OR: 0.36, 95% CI: 0.13–0.93). For MDD, PCL total scores (OR: 1.17, 95% CI: 1.11–1.23), male gender (OR: 6.12, 95% CI: 1.70–21.95), previous combat exposure (OR: 6.38, 95% CI: 1.17–34.91) and Type D personality (OR: 10.81, 95% CI: 2.78–41.98) were positive predictors.

### Neurocognitive performance

The sample was stratified based on self-reported stress-related mental health symptoms, resulting in PTSD+ (*n* = 18), MDD+ (*n* = 38), Co-Morbid+ (*n* = 43), and Asymptomatic (*n* = 98) symptom groups. Evaluation of MANCOVA model assumptions revealed no significant deviations from normality and homogeneity of variance-covariance matrices. Results of the MANCOVA revealed a statistically significant multivariate effect of Symptom Groups according to Pillai's Trace (0.25), *F*_(27, 552)_ = 1.84, *p* = 0.006, η^2^ = 0.083. To distill the linear combination of DANA battery assessments contributing to the significant multivariate effect, descriptive discriminant analysis was used as a follow-up analysis to the MANCOVA. A single, statistically significant function was extracted (Wilks λ = 0.775, $\chi_{\left(27\right)}^{2}$ = 50.11, *p* = 0.004), which accounted for 70.4% of the variance in Symptom Groups. Adjusted means and standard deviations, as well as standardized canonical discriminant function coefficients for each neurocognitive assessment are reported in Table 5. The coefficients indicated greater weighting of simple reaction time (second administration; 0.533) and go/no-go (0.741) in maximizing differences between Symptom Groups. A simplified multivariate composite score was thus derived from the descriptive discriminant analysis, which we conceptually framed within the domain of motor speed and termed *Cognitive Motor Speed*. To further delineate neurocognitive performance within Symptom Groups, Cognitive Motor Speed was entered as a dependent variable in a univariate ANCOVA, using Symptom Groups as the independent variable and adjusting for age, gender, and number of previous concussions. The results of this analysis indicated a main effect of Symptom Groups, *F*_(3, 190)_ = 10.54, *p* < 0.001, η^2^ = 0.14. Bonferroni-corrected *post-hoc* pairwise comparisons revealed the Co-Morbid+ symptom group (*M* = 90.00, *SE* = 2.87) demonstrated significantly worse overall performance compared to MDD+ (*M* = 106.18, *SE* = 3.15), PTSD+ (*M* = 109.08, *SE* = 4.47), and Asymptomatic (*M* = 108.63, *SE* = 1.92) groups (all *p*'s < 0.01).

**Table 5:** Means, standard deviations, and discriminant function coefficients for symptom groups on neurocognitive assessments.

	**SYMPTOM GROUP**				
**DANA assessment**	**Asymptomatic (*n* = 98)**	**PTSD+ (*n* = 18)**	**MDD+ (*n* = 38)**	**Co-Occurring+ (*n* = 43)**	***w_*s*_***
SRT1 TP	185.88 (24.96)	172.62 (24.08)	178.72 (33.44)	180.66 (28.74)	−0.418
SRT2 TP	189.87 (25.16)	179.07 (25.02)	184.06 (24.54)	170.95 (28.84)	0.533
CS-L TP	44.38 (9.03)	43.96 (6.45)	43.68 (8.98)	41.36 (8.07)	0.021
CS-R TP	47.08 (13.88)	47.35 (9.11)	47.52 (12.43)	43.52 (11.51)	0.145
PRT TP	97.31 (13.60)	98.03 (14.03)	95.84 (12.67)	91.36 (15.13)	0.105
SPD TP	29.87 (6.98)	30.04 (6.24)	29.96 (6.02)	30.27 (6.33)	−0.430
GNG TP	118.77 (16.33)	117.85 (14.46)	115.28 (13.02)	105.47 (18.36)	0.741
MTS TP	32.40 (8.24)	28.70 (7.50)	31.60 (8.94)	29.86 (8.43)	0.220
SMS TP	54.70 (17.18)	47.89 (18.82)	55.65 (20.17)	51.36 (19.50)	−0.273

*TP, Throughput; SRT1, Simple Reaction Time 1; SRT2, Simple Reaction Time 2; CS-L, Code Substitution Learning; CS-R, Code Substitution Recall; PRT, Procedural Reaction Time; SPD, Spatial Discrimination; GNG, Go/No-Go; MTS, Match to Sample; SMS, Sternberg Memory Search. ws, standardized discriminant function coefficient. Values represent average TP scores and standard deviations*.

## Discussion

Coast Guard personnel serving at Boat Stations experience shift duty with a mixture of operational and off-duty civilian stressors. Overall caseness for PTSD in this sample of CG personnel was similar to caseness using PCL-S (not specific to source) or PCL-C in large cross-sectional studies of Army (Hoge et al., 2008, 2014; Vasterling et al., 2010; Sundin et al., 2014), Marine (Hoge et al., 2014), and Navy (Harbertson et al., 2016) active duty personnel. Rates of PTSD were also consistent with cross-sectional studies of law enforcement (Maia et al., 2008; Bowler et al., 2010), civilian first responders (Skogstad et al., 2015) and firefighters in cross-sectional studies using similar instruments.

To capture the source of stress-related mental health difficulties, CG personnel were administered both the PCL-M and PCL-NM. Comparisons between the two scales suggest that CG personnel adhered to the difference in prompts and responded with different traumas in mind. The delivery of two PCLs is rarely reported, however, Hoge and colleagues recently reported on a comparison between the PCL-S which is based on DSM-IV criteria and the PCL-5, which is based on DSM-5 criteria, in a military sample. A high degree of agreement (κ = 0.67) was obtained with the PCL-S with PCL-5 using the same stressor prompt (Hoge et al., 2014). This contrasts to the modest agreement (κ = 0.37) obtained herein with two different prompts. Caseness of PTSD from non-military traumas was roughly double caseness from military trauma when accounting for personnel that met criteria on both versions. The bias toward non-military traumas may be attributable to: (a) less exposure to military traumas compared to non-military traumas, (b) greater proportion of time CG personnel spend engaged in off-base activities and non-military life events, or (c) an indirect effect of military traumas which depletes resources necessary to cope with non-military traumas. As to the former, there is no validated instrument to assess traumas encountered by CG personnel in performance of duty similar to the CES. Future work is necessary to more fully detail rates and sources of trauma in relation to PTSD symptoms. An open question arising from the cross sectional rates is whether enduring PTSD is more related to military, non-military or the combined burden. This question may be extended to hazardous civilian occupations.

Rates of MDD using symptom criteria were consistent with large surveys of active duty military (Wells et al., 2013), but half of what has been reported to post-deployed forces (Luxton et al., 2010). Further, the degree of co-occurrence of MDD and PTSD was similar to that observed in large survey studies (Wilk et al., 2012).

With the relatively large sample of females, gender as an influence on PTSD and MDD was evaluated. Rates of PTSD were equivalent in male and female CG personnel regardless of screening criteria or type of trauma experienced. Although, females are regarded as having higher risk of PTSD within the general population, studies of active duty military are mixed (Crum-Cianflone and Jacobson, 2014), or do not find differences (Jacobson et al., 2015). A lack of difference has also been reported in studies of police officers (Pole et al., 2001), whose duty demands are similar between males and females. Being female was a predictive factor for MDD when more liberal aggregate scoring criteria were imposed and attendant mild depression was included in models. The difference may be attributed to poor sleep quality which contributes to MDD symptoms, especially mild depression, and was more prevalent in females.

An important objective of the current study was to assess the ability of two personality factors previously identified as vulnerabilities—BI and Type D—to predict stress-related mental health problems. Consistent with research in veterans (Myers et al., 2012a,b), BI temperament was strongly associated with PTSD. BI temperament significantly classified subclinical and full PTSD emanating from either military or non-military traumas. However, BI temperament was not a significant predictor of MDD. In contrast, Type D personality strongly predicted MDD, but was not predictive of PTSD. Given the emphasis of Type D on negative affect it is not surprising that Type D is predictive of depressive mood. Although temperamental dispositions are considered stable personality characteristics, the cross-sectional nature of the study precludes stronger statements regarding either personality factor as a vulnerability to development PTSD or MDD.

There has been growing concern that mTBI may have long-term adverse mental and cognitive consequences. Although roughly half the sample screened positive for lifetime mTBI, lifetime mTBI or the number of mTBI events encountered were not predictors of PTSD, MDD or any deficits in neurocognitive performance.

Neurocognitive performance was, however, distinguishable based on caseness for mental health outcomes. Impaired neurocognitive performance was concentrated among those expressing caseness for both PTSD and MDD. The poor performance was mostly driven by deficits in simple reaction time and response inhibition. The DANA neuropsychological tests are subset of those comprising the Automated Neurobehavioral Assessment Metric (ANAM), but on a hand held form factor. Previous studies with active duty military assessing ANAM performance found deficits in simple reaction time (Vasterling et al., 2012; Wilson et al., 2016), code substitution learning and recall (Vasterling et al., 2012), code substitution learning, and match to sample (Neipert et al., 2014) in those meeting criteria for PTSD. Depressive symptoms were not evaluated in these studies. In these studies, the response inhibition module (“Go/No Go”) was not administered. Response inhibition is deficient in civilians (Falconer et al., 2013) and veterans with PTSD (Swick et al., 2012, 2013). Sensitivity to neurocognitive deficits in PTSD and MDD may depend on a number of factors, including form and venue of testing, and population size. To what degree the reduced throughput on these cognitive tasks adversely affects operational effectiveness of active military personnel, particularly decision making under stress, is unknown.

The main strength of the study is the examination of otherwise healthy high-functioning active duty CG personnel, an underrepresented military population. Not one participant was on reduced or modified duty. Another strength was the number of female volunteers, which allowed for assessments of the influence of gender in many of the outcomes. However, there are several weaknesses that need to be considered. For one, the sample was neither representative of all CG Boats Stations or of the general CG force. For another, the cross-sectional nature of assessment precludes sensitivity to fluctuations in emotional and cognitive performance. Although personality tendencies are assumed to be stable individual characteristics, the nature of the study does not preclude the possibility that experiences such as head injury or those leading to stress-related mental health symptoms can concomitantly change personality characteristics. Further, as a convenience sample of CG Boat Stations, many of the cofactors considered (e.g., sex, deployment, CES) were unbalanced and resulted in small numbers when trying to identify interactions. For example, females in the sample were generally younger and concentrated in lower ranks than males. Boat Stations are heterogeneous in military roles (boat crews, engineers, cooks, administrative, and support staff) and shifts. The inclusiveness of the study to all willing participants comes at the expense of focus on particular operational roles, which would require a much larger study to capture. Additionally, testing was conducted on an individualized basis, therefore time of testing—within a range of 700 to 1,700—was not controlled. Volunteers were allowed to schedule testing when testing would not interfere with duty. Therefore, control was also not exerted in terms of type of experiences immediately preceding testing (e.g., operations, exercise, meals, or training).

To date, this is the largest empirical study of stress and stress-related mental symptoms in CG personnel. The rates of PTSD related to military and non-military experiences are provocative, generating questions for future studies. Accounting for personality could provide opportunities for early intervention, promoting resiliency or targeted tracking to reduce expression of PTSD and/or MDD in the aftermath of trauma.

## Author contributions

RS was responsible for study design, implementation, consenting participants, testing, study oversight, data analysis, and dissemination. JH was responsible for data analysis, write up, and dissemination. MD was responsible for study design, implementation and oversight. JT, CaEM, ChEM, RL, and WW were responsible for study design, development of tools and instruments for the conduct of the study, data analysis, write up and dissemination. PA and NK were responsible for consenting participants, testing and data entry. All authors contributed to and have approved the final manuscript.

### Conflict of interest statement

The authors declare that the research was conducted in the absence of any commercial or financial relationships that could be construed as a potential conflict of interest.
